# A Poly-Lysine-Based RBD Mucosal Vaccine Induces Potent Antibody Responses in Mice

**DOI:** 10.3390/vaccines13060582

**Published:** 2025-05-29

**Authors:** Huifang Xu, Han Wang, Peng Sun, Tiantian Wang, Bin Zhang, Xuchen Hou, Jun Wu, Bo Liu

**Affiliations:** 1 Laboratory of Advanced Biotechnology, Beijing Institute of Biotechnology, 20 Dongdajie Street, Fengtai District, Beijing 100071, China; xuhuifang9169@163.com (H.X.); wanghan010811@163.com (H.W.); sunpeng990718@163.com (P.S.); con_an@126.com (T.W.); zhangbin@bmi.ac.cn (B.Z.); hoxuch@163.com (X.H.); 2 School of Basic Medical Sciences, Tsinghua University, No. 30 Shuangqing Road, Haidian District, Beijing 100084, China

**Keywords:** SARS-CoV-2, RBD, poly-lysine, mucosal vaccine, subunit vaccine

## Abstract

(1) Background: The COVID-19 pandemic highlights the critical necessity for the development of mucosal vaccines. (2) Objective: In this study, we aimed to develop mucosal vaccines based on the receptor-binding domain (RBD) of the SARS-CoV-2 Spike protein. (3) Methods: We engineered the RBD of the Spike protein by incorporating ten lysine residues (K10), thereby enhancing its positive charge under physiological conditions. (4) Results: Although this modification did not directly augment the immunogenicity of the antigen, its combination with the mucosal adjuvant cholera toxin B subunit (CTB) and administration via the pulmonary route in BALB/c mice resulted in the induction of robust neutralizing antibody titers. Antigen-specific antibody responses were observed in both serum and bronchoalveolar lavage fluid. Importantly, serum IgG antibody titers remained above 10^4^ six months following third immunization, suggesting the establishment of sustained long-term immunity. Additionally, the incorporation of five lysine residues (K5) into the RBD, in conjunction with CTB, significantly increased serum IgG and IgA antibody titers. (5) Conclusions: Adding poly-lysine to RBD and combining it with CTB can stimulate robust mucosal and humoral immune responses in mice. These findings offer valuable insights for the design of subunit mucosal vaccines.

## 1. Introduction

Coronavirus disease 2019 (COVID-19), instigated by the severe acute respiratory syndrome coronavirus 2 (SARS-CoV-2), was officially designated as a pandemic in 2020 [1]. Vaccination has emerged as a highly efficacious public health strategy for preventing and controlling the dissemination of this debilitating respiratory syndrome. A variety of SARS-CoV-2 vaccine formulations, including inactivated [2], subunit [3], mRNA [4], and viral vector vaccines [5], have attained regulatory approval. Predominantly, these vaccines are administered via intramuscular injection, which induces the production of serum immunoglobulin (Ig) G and neutralizing antibodies. Nonetheless, a limitation of this approach is the lack of mucosal IgA and IgG, as intramuscular vaccination does not establish a primary defense mechanism within the respiratory tract.

Numerous mucosal vaccine candidates targeting SARS-CoV-2 are presently under development or have received authorization [6], with the majority employing vectors derived from adenoviruses, influenza viruses, or parainfluenza viruses. The efficacy of vaccines based on viral vectors may be compromised by the presence of pre-existing immunity [7]. Subunit vaccines, which are characterized by their well-defined components and highly specific immunogenicity, continue to hold significant promise for future development [8,9]. Nevertheless, vaccines based on the Spike (S) protein of SARS-CoV-2 may also experience reduced efficacy due to immune imprinting [10,11]. The receptor-binding domain (RBD), recognized for its ability to bind to angiotensin-converting enzyme 2 (ACE2) [12] and its inclusion of the majority of neutralizing epitopes, is regarded as a critical target for the development of subunit vaccines [13,14].

Mucosal subunit vaccines face significant challenges in eliciting antigen-specific immune responses due to the mucosal barrier, which impedes the delivery of antigens to antigen-presenting cells (APCs) such as dendritic cells (DCs), macrophages, and B cells. Two primary obstacles exist in the delivery of antigens to APCs in mucosal vaccines. The first is the mucosal epithelial barrier, which prevents antigens from entering the body [15]. Tight junctions within the mucosal epithelium, along with ciliary motion, impede vaccine uptake, diminish retention time, and facilitate rapid clearance from the body [16,17]. Additionally, even if antigens successfully traverse this initial barrier, they must be efficiently delivered to and activate APCs to initiate robust antigen-specific immune responses. The activation of APCs is crucial for the induction of adaptive immune responses. Mucosal adjuvants play a critical role in enhancing antigen presentation and activating APCs, thereby facilitating the development of antigen-specific immune responses [18,19].

To improve antigen retention at mucosal surfaces, research has utilized the positive charge of chitosan to engage in electrostatic interactions with the negatively charged mucosal membranes, thereby developing RBD-based mucosal vaccines. Intranasal administration of these vaccines in murine models has elicited robust cross-reactive immune responses, indicating that positively charged antigens may enhance mucosal immunity [20]. Another study employed a series of intricate procedures to develop an RBD vaccine, utilizing the principle of electrostatic attraction between positive and negative charges by integrating RBD, polyarginine, mannan, and 2′3′-cGAMP. This design is challenged by its complexity in preparation, and the reliance on electrostatic adsorption raises the potential for desorption during immunization. Consequently, the antigen and adjuvant may not target the same cell, leading to suboptimal immune responses. Nevertheless, this study highlights the potential for optimizing antigens by leveraging their charge properties. The cholera toxin B subunit (CTB), a well-recognized mucosal adjuvant, is known to augment antigen presentation and immune activation. Moreover, engineered CTB nanoparticles, in conjunction with biodegradable polymer encapsulation, have demonstrated enhanced mucosal immune activation in SARS-CoV-2 mucosal vaccine formulations [21].

**Figure 1:**
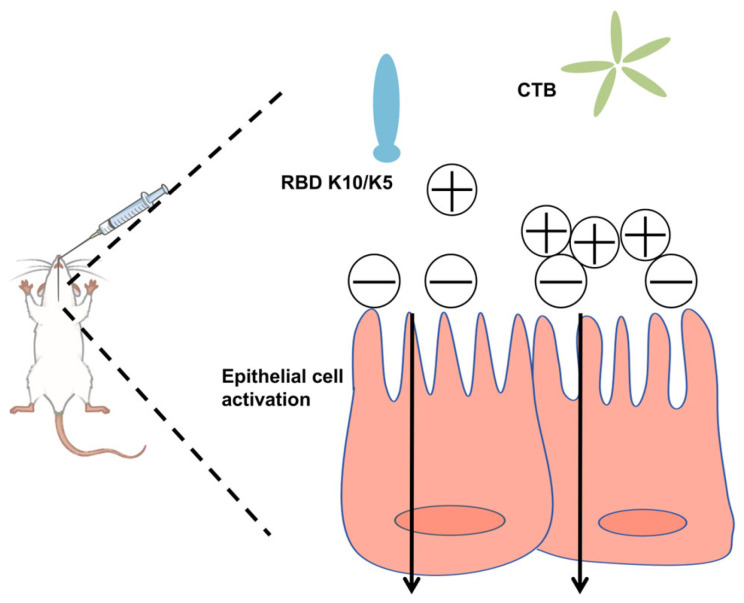
Schematic illustration of the vaccine design strategy.

In this study, we postulated that augmenting the positive charge of an antigen under physiological conditions would facilitate its electrostatic adsorption to negatively charged mucosal surfaces, thereby extending its retention time. To evaluate this hypothesis, we increased the net positive charge of the RBD by incorporating ten lysine (K10) residues at its C-terminus. This modification conferred a positive charge to the protein under physiological conditions. To mitigate the weak immunogenicity typically associated with subunit mucosal vaccines, we utilized the CTB as an effective mucosal adjuvant (Figure 1). The findings revealed that the mucosal vaccine induced robust humoral immune responses in mice, demonstrating significantly enhanced neutralizing activity against a pseudovirus compared to the native RBD.

## 2. Materials and Methods

### 2.1. Glycoengineered Pichia Pastoris, Bacterial Strains, and Materials

The glycoengineered strain of *Pichia pastoris* was maintained in our laboratory under controlled conditions. Cultivation of *P. pastoris* strains was performed in yeast peptone dextrose (YPD) medium at a temperature of 25 °C. *Escherichia coli* Trans5α (TransGen Biotech, Beijing, China) was cultured in Luria–Bertani medium at 37 °C. The yeast extract, agar, and tryptone utilized in these processes were obtained from OXOID (Basingstoke, UK), while NaCl was sourced from SINOPHARM (Shanghai, China). Additionally, Zeocin was supplied by Thermo Fisher Scientific (Waltham, MA, USA).

### 2.2. Construction of the RBD K10 and RBD K5 Expression Vectors and Screening for Positive Clones

The plasmid pPICZαA-RBD was engineered and preserved within our laboratory, as previously described [22]. To develop RBD expression vectors incorporating poly-lysine tags, sequences encoding K10 and K5 were appended to the C-terminus of the RBD sequence (wild-type) and inserted into the *Xho*I and *Sac*I restriction sites of the pPICZαA vector, resulting in the construction of plasmids pPICZαA-RBD K10 and pPICZαA-RBD K5. These plasmids were linearized using *Bgl*II and subsequently transformed into glycoengineered *Pichia pastoris*. Protein expression was induced with methanol, and positive clones were identified via Western blot analysis employing anti-his tag antibodies conjugated with horseradish peroxidase (HRP) at a dilution of 1:2500 (Sigma-Aldrich, Taufkirchen, Germany).

### 2.3. RBD K10 and RBD K5 Protein Purification

Clones confirmed as positive by Western blot analysis were subjected to scale-up cultivation in shake flasks. Following methanol induction for 72 h, the culture was centrifuged at 8000× *g* for 15 min to harvest the supernatant. The supernatant was diluted twofold with water and purified through gradient elution using Ni Sepharose 6 Fast Flow (GE Healthcare, Chicago, IL, USA). The purified protein underwent further concentration by ultrafiltration and was buffer-exchanged into 0.9% NaCl for subsequent applications. The purified protein underwent analysis via 12% sodium dodecyl sulfate-polyacrylamide gel electrophoresis (SDS-PAGE).

### 2.4. Structural Characterization and Identification of the RBD K10 Protein

The purified RBD K10 protein was subjected to treatment with peptide-N-glycosidase F (PNGF, maintained in our laboratory) at 37 °C for a duration of 12 h. Post-treatment, the samples were analyzed using 12% SDS-PAGE and Western blot techniques. For the Western blot analysis, an anti-his tag (HRP) primary antibody was employed at a dilution ratio of 1:2500.

### 2.5. RBD-K10 Vaccine Formulation and Immunization Detection

Female BALB/c mice, aged 6–8 weeks, were procured from Beijing Weitonglihua Laboratory Animal Technology Co., Ltd. (Beijing, China), and housed at the Animal Center of the Beijing Institute of Biotechnology (Beijing, China). The experimental protocol was approved under the animal welfare ethics number IACUC-2024-004 on 25 January 2024. Cholera toxin B (CTB) was sourced from Beyotime Biotechnology (Shanghai, China). The pulmonary delivery device was obtained from Beijing Huironghe Technology (Beijing, China), and the mouse restraint agent, tribromoethanol, was purchased from Beijing Lai Aite Technology Development Co., Ltd. (Beijing, China). To evaluate the efficacy of the mucosal vaccine, mice were randomly assigned to the following immunization groups, with each group comprising 10 mice: (1) saline; (2) 10 μg RBD; (3) 10 μg RBD K10; (4) 10 μg RBD + 2 μg CTB; (5) 10 μg RBD K10 + 2 μg CTB. Prior to pulmonary immunization, the mice received an intraperitoneal injection of tribromoethanol in accordance with the manufacturer’s guidelines. The vaccines were then administered via a pulmonary delivery device, with each mouse receiving a dose of 50 μL. Immunizations were conducted on days 0, 14, and 28, resulting in a total of three immunization sessions. Serum samples were collected prior to each immunization, and bronchoalveolar lavage fluid (BALF) was obtained two weeks following the third immunization.

### 2.6. ELISA (Enzyme-Linked Immunosorbent Assay)

The RBD was diluted to a concentration of 2 μg/mL using a coating buffer composed of 50 mmol/L carbonate at pH 9.6. Subsequently, 100 μL of this solution was added to each well of ELISA plates (Corning, NY, USA, 3590), which were then stored at 4 °C overnight. Following incubation, the plates underwent two washes with PBST (phosphate-buffered saline with 0.1% Tween-20). Each well was then treated with 300 μL of 5% skimmed milk and incubated for 1 h at 37 °C. Serum, diluted in a gradient, was applied to an ELISA plate at a volume of 100 μL per well. The plates were incubated at 37 °C for 1 h and washed three times with PBST. Subsequently, 100 μL of goat anti-mouse IgG (HRP) antibody, diluted 1:4000 (Beijing Biodragon Immunotechnologies, Beijing, China, BF03112), was added to each well, followed by a 1 h incubation at 37 °C. For serum antibody isotyping, IgG1 (Abcam, Cambridge, MA, USA, ab97240) and IgG2a (Abcam, ab97245) antibodies were introduced at a 1:5000 dilution. For IgA detection, the secondary antibody was replaced with an IgA secondary antibody (Abcam, ab97235) at a 1:8000 dilution. The plates were subsequently washed four times with PBST and developed using 100 μL per well of TMB one-component chromogenic solution (Solarbio, Beijing, China, PR1200) for a duration of 4 min. To halt the reaction, 50 μL per well of 2 M H_2_SO_4_ was added. The results were measured using a microplate reader set at 450 nm.

### 2.7. Pseudovirus Packaging

HEK293T cells, maintained in our laboratory, underwent two washes with PBS, followed by aspiration of any residual liquid. Subsequently, 1 mL of trypsin (HyClone, Logan, UT, USA, SV30031.01) was introduced to digestion, which was then terminated by the addition of 3 mL of complete DMEM medium (comprised 89% DMEM (Gibco, Waltham, MA, USA, C11995500BT), 10% fetal bovine serum (PAN-Biotech, Aidenbach, Bayern, Germany, P30-3306), and 1% penicillin–streptomycin (Gibco, 15140122)). A total of 12 mL of cells were seeded at a density of 5 × 10^5^ cells/mL into 10 cm dishes (Corning, 430167). The cells were incubated at 37 °C in a 5% CO_2_ incubator for 12 h. Prior to transfection, the growth status of the cells was evaluated. In accordance with the Lipofectamine 3000 transfection reagent protocol (Thermo Fisher Scientific, L3000015), 7.5 μg of the pNL4-3-R-E backbone plasmid (from laboratory stock) and 7.5 μg of the SARS-CoV-2 S protein plasmid (synthesized by Sangon Biotech, Shanghai, China) were transfected. Six to eight hours post-transfection, the medium was replaced with Opti-MEM (Gibco, 31985070). At 72 h post-transfection, the supernatant was collected by centrifugation at 1000× *g* for 5 min, filtered through a 0.45 μm membrane, and stored at –80 °C for future use.

### 2.8. Pseudovirus Neutralization Assay

HEK293T-ACE2 cells (maintained in our laboratory) were seeded one day prior at a density of 2 × 10^4^ cells per well in cell culture plates (Corning, 3599). The serum was pre-inactivated by heating at 56 °C for 30 min. A volume of 150 μL of diluted serum was added to the first well in each row, followed by the addition of 50 to 100 μL of medium to the subsequent wells in a three-fold serial dilution, resulting in a total of five dilutions (including the first well). Medium was added to the virus control wells (100 μL) and the cell control wells (150 μL). Pseudoviruses were diluted to a concentration of 2 × 10^4^ TCID50/mL, and 50 μL was added to each of the sample and virus control wells. The mixture was shaken and mixed for 2 min, followed by neutralization at 37 °C for 1 h. Subsequently, the cell culture medium was removed, and 120 μL of the serum–virus mixture was transferred to plates containing cells. After an incubation period of 8 h, the medium in the plates was aspirated, and 150 μL of fresh medium was added. The cells were then cultured for an additional 48 h. Subsequently, 100 μL of medium was removed from each well, and 100 μL of reporter gene assay reagent (Vazyme, Nanjing, Jiangsu, China, DD1201) was added. A total of 100 μL of the reaction mixture was then transferred to a CulturPlate-96 white plate (PerkinElmer, Waltham, MA, USA, 6005290), and fluorescence values were measured using an enzyme marker.

### 2.9. Statistical Analysis

Data were processed and visualized using GraphPad Prism 10.3.0 software. For the analysis of multiple parallel groups, a one-way analysis of variance (ANOVA) with Dunnett’s multiple comparison test was conducted. In cases where the experimental design included two variables, a two-way ANOVA was employed for multiple comparison analysis. Statistical significance was defined as follows: not significant difference (ns), *p* > 0.05; * *p* < 0.05; ** *p* < 0.01; *** *p* < 0.001; **** *p* < 0.0001. All results are presented as mean ± standard deviation (Mean ± SD).

## 3. Results

### 3.1. RBD K10 Expressed by Glycoengineered P. pastoris Has N-glycosylation Modification

**Figure 2:**
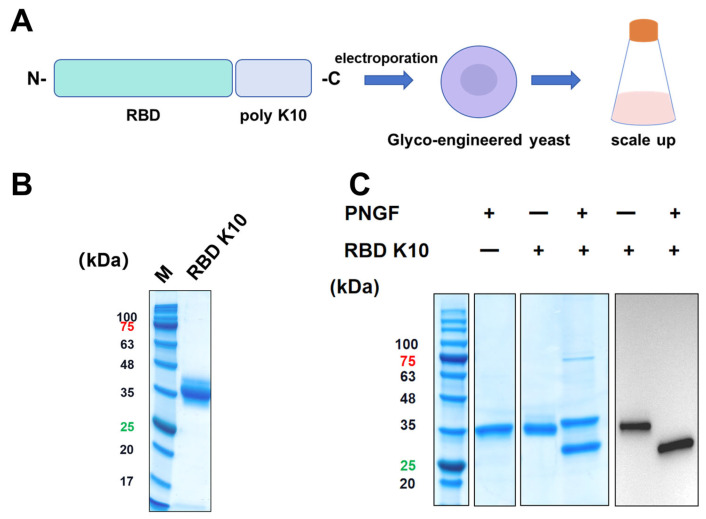
Illustrates the design, expression, and analysis of the recombinant RBD K10 protein. (**A**) Schematic representation of the recombinant RBD K10 construct. A poly-lysine (K10) tag is fused to the receptor-binding domain (RBD) at the C-terminus. The construct was introduced into glycoengineered *Pichia pastoris* via electroporation. Positive clones were subsequently screened and subjected to scale-up cultivation. (**B**) SDS-PAGE analysis of the purified RBD K10 protein. The molecular weight marker (M) is shown on the left, and the purified RBD K10 protein appears as a single major band. (**C**) SDS-PAGE and Western blot analysis of the undigested or PNGF-digested RBD K10 protein. These numbers indicate the molecular weights of proteins, with red indicating a protein molecular weight of 75 kDa and green indicating a molecular weight of 25 kDa.

To impart a positive charge to the RBD under physiological conditions, ten lysine residues were introduced at the C-terminus, resulting in the RBD K10. This newly engineered RBD was subsequently expressed using a glycoengineered *P. pastoris* expression system previously developed by our team (Figure 2A) [23]. Positive clones were identified via Western blot screening. The target protein, RBD K10, was purified and subjected to analysis by SDS-PAGE, as depicted in Figure 2B. Consistent with prior findings, the RBD K10 expressed in glycoengineered *P. pastoris* displayed a molecular weight of approximately 35 kDa prior to PNGF digestion. Upon enzymatic cleavage, the band shifted significantly downward to approximately 27 kDa (Figure 2C), aligning with its theoretical molecular weight in the non-glycosylated state. Western blot analyses further corroborated the presence of glycosylation modifications, specifically indicating N-glycosylation of the target antigen.

### 3.2. RBD K10 and CTB-Enhanced RBD-Specific IgG Response via Pulmonary Delivery in Mice

**Figure 3:**
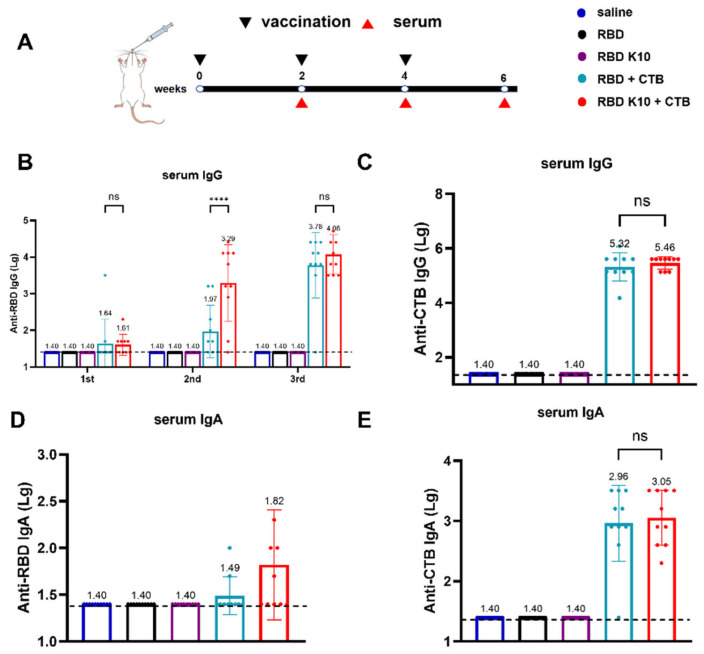
Immunogenicity evaluation of RBD K10 and its combination with and without cholera toxin B subunit (CTB) in mice following pulmonary delivery. (**A**) Experimental timeline depicting vaccination (black triangles) and serum collection (red triangles) over a 6-week period. Mice were immunized via pulmonary delivery with saline, RBD, RBD K10, RBD + CTB, or RBD K10 + CTB. (**B**) Serum anti-RBD IgG levels at weeks 2, 4, and 6. (**C**) Anti-CTB IgG levels in serum at week 6. (**D**) Serum anti-RBD and (**E**) anti-CTB IgA levels at week 6. (*n* = 10). not significant difference (ns) *p* > 0.05; **** *p* < 0.0001.

To assess the immunogenicity of RBD K10, particularly in light of the potentially low immunogenicity associated with RBD, the mucosal adjuvant CTB was incorporated into the study. Mice were immunized via pulmonary delivery on days 0, 14, and 28, with blood samples collected two weeks following each immunization to evaluate serum antibody titers (Figure 3A). The serum titers of RBD-specific IgG antibodies were quantified, as depicted in Figure 3B. No detectable antibodies were observed in the saline, RBD, and RBD K10 groups. In contrast, the RBD + CTB group exhibited a low titer two weeks after the initial immunization, which increased to 1:93 following the second immunization, a level significantly lower than that observed in the RBD K10 + CTB group (1:1950, *p* < 0.0001). After the third immunization, titers reached 6.03 × 10^3^ and 1.20 × 10^4^ in the RBD + CTB and RBD K10 + CTB groups, respectively. CTB-specific IgG antibody titers, measured two weeks post-third immunization (Figure 3C), were undetectable in the saline, RBD, and RBD K10 groups, whereas no significant difference was observed between the RBD + CTB and RBD K10 + CTB groups. The levels of serum RBD-specific IgA antibodies following the third immunization are presented in Figure 3D. No measurable titers were observed in the saline, RBD, and RBD K10 groups. In contrast, the RBD + CTB and RBD K10 + CTB groups exhibited titers of 1:31 and 1:66, respectively, with the latter group demonstrating a slightly elevated titer. Additionally, CTB-specific IgA levels did not reveal any significant differences between the two CTB-supplemented groups (Figure 3E). These results underscore the efficacy of CTB in significantly enhancing humoral immunity, particularly when combined with RBD K10, which resulted in even higher antibody titers.

### 3.3. RBD K10 + CTB Induces Diverse IgG Antibody Subtypes

**Figure 4:**
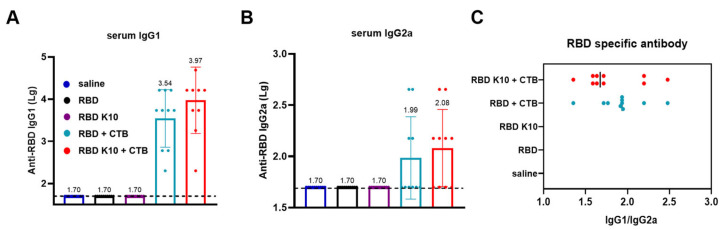
Characterization of serum antibody subclasses following the third immunization with RBD K10. (**A**) Serum IgG1 and (**B**) IgG2a antibody titers two weeks after the third immunization. (**C**) The ratio of IgG1 to IgG2a. (*n* = 10).

IgG1 and IgG2a, which are subclasses of IgG, are differentially regulated by T-helper cell cytokines. The Th2 cytokine IL-4 stimulates B-cell production of IgG1 while concurrently suppressing IgG2a secretion. Conversely, the Th1 cytokine IFN-γ promotes IgG2a synthesis and inhibits IgG1 production. The distinct functions of IgG1 and IgG2a illustrate the immune system’s ability to balance antibody responses (IgG1) with inflammatory responses (IgG2a), thereby enabling a tailored defense mechanism against various pathogens [24]. In the group immunized with RBD + CTB, IgG1 antibody titers reached 3.47 × 10^3^ (Figure 4A), while IgG2a titers were 1:98 (Figure 4B). In contrast, the RBD K10 + CTB group demonstrated higher titers, with IgG1 at 9.33 × 10^3^ and IgG2a at 1.20 × 10^2^. In the RBD K10 + CTB group, the serum IgG1/IgG2a ratio approximated 1, suggesting a predisposition towards cellular immunity (Figure 4C). These findings indicate that the combination of RBD and CTB effectively induces a diverse antibody response, predominantly IgG1. Moreover, when compared to the native RBD, the modified RBD K10, in conjunction with CTB, elicits a more robust immunostimulatory effect, enhancing both IgG1 and IgG2a antibody titers.

### 3.4. RBD K10 + CTB Stimulates Strong Mucosal Immunity in BALF of Mice Administered by Pulmonary Delivery

**Figure 5:**
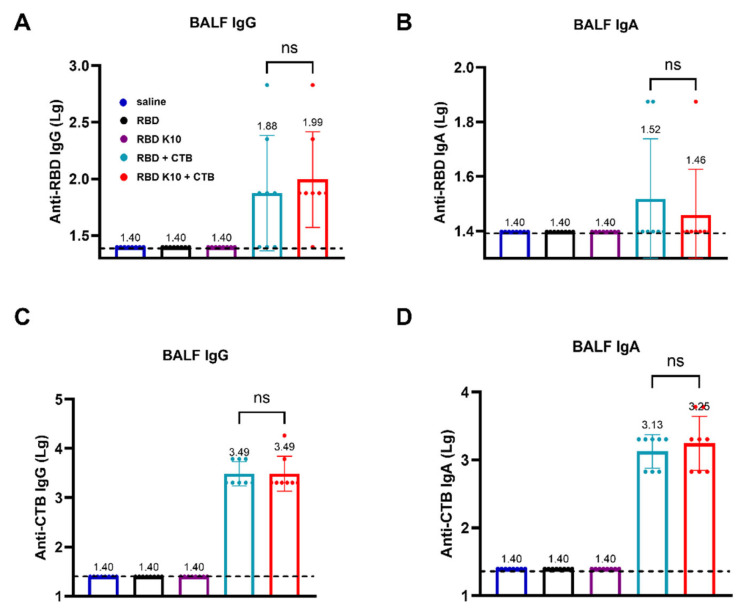
Evaluation of mucosal immune responses in bronchoalveolar lavage fluid (BALF) from mice immunized RBD K10 via pulmonary delivery. (**A**) Anti-RBD IgG and (**B**) IgA levels in BALF. (**C**) BALF anti-CTB IgG and (**D**) IgA levels. ns, *p* > 0.05.

To evaluate whether pulmonary delivery of RBD K10 and related formulations induces a mucosal immune response in mice, we measured RBD- and CTB-specific IgG and IgA antibody titers in bronchoalveolar lavage fluid (BALF). RBD-specific IgG antibody titers in BALF were assessed, with results depicted in Figure 5A. No detectable titers were observed in the saline, RBD, and RBD K10 groups, whereas the RBD + CTB and RBD K10 + CTB groups exhibited titers of 1:76 and 1:98, respectively. In contrast, RBD-specific IgA antibody titers remained low across all groups (Figure 5B). In a similar vein, the titers of CTB-specific IgG antibodies in BALF were assessed (Figure 5C), demonstrating equivalent titers of 3.09 × 10^3^ in both the RBD + CTB and RBD K10 + CTB groups. In terms of CTB-specific IgA antibodies, the titers were measured at 1.35 × 10^3^ and 1.78 × 10^3^ for the RBD + CTB and RBD K10 + CTB groups, respectively, with no statistically significant differences observed between the two (Figure 5D). These results suggest that pulmonary administration of RBD + CTB and RBD K10 + CTB effectively elicits mucosal antibody responses in BALF, characterized by comparable CTB-specific titers and a modest induction of RBD-specific IgA.

### 3.5. RBD K10 + CTB Triggers Robust Neutralizing Antibody Titers

**Figure 6:**
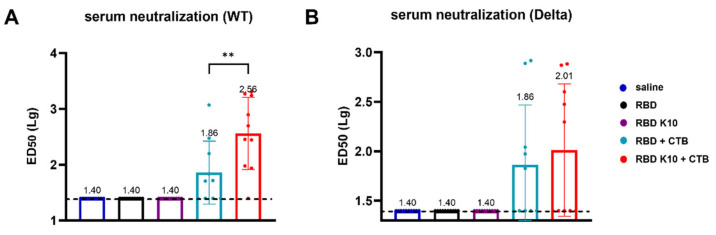
Serum neutralizing antibody activity following pulmonary delivery of RBD K10. (**A**) Neutralizing antibody titers against SARS-CoV-2 wild-type and (**B**) Delta variant pseudovirus in serum of mice immunized via pulmonary delivery of RBD K10. (*n* = 10) ED50: median effective dose. ** *p* < 0.01.

To further evaluate the efficacy of RBD K10 and related formulations following pulmonary delivery, we examined serum neutralizing activity. No neutralizing antibodies were detected in the saline, RBD, and RBD K10 groups against the wild-type SARS-CoV-2 pseudovirus. In contrast, the RBD K10 + CTB group exhibited a neutralizing antibody titer of 1:363, which was significantly higher than the 1:72 observed in the RBD + CTB group (*p* < 0.01) (Figure 6A). For the Delta variant pseudovirus, the neutralizing antibody titer in the RBD K10 + CTB group reached 1:102, exceeding the 1:72 observed in the RBD + CTB group (Figure 6B). These results indicate that the combination of RBD with CTB significantly enhances serum neutralizing antibody titers, with the incorporation of 10 lysine residues in RBD K10 further augmenting this response.

### 3.6. RBD K10 + CTB Triggers Durable Immune Responses

**Figure 7:**
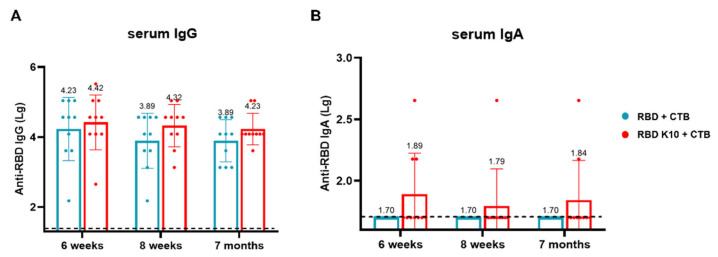
Long-term efficacy evaluation of RBD K10 via pulmonary delivery. (**A**) Long-term RBD-specific IgG and (**B**) IgA antibody titers in the serum of mice immunized via pulmonary delivery of RBD K10 at 6 weeks, 8 weeks, and 7 months.

To evaluate the long-term immune efficacy of this combination following pulmonary administration, blood samples were collected, and serum was isolated from mice at two weeks, four weeks, and six months after the third immunization to measure IgG and IgA antibody titers. In the RBD K10 + CTB group, RBD-specific IgG antibody titers were 2.63 × 10^4^, 2.09 × 10^4^, and 1.70 × 10^4^ at two weeks, four weeks, and 7 months, respectively, whereas the RBD + CTB group exhibited titers of 1.70 × 10^4^, 7.76 × 10^3^, and 7.76 × 10^3^ (Figure 7A). Notably, no RBD-specific IgA antibodies were detected in the RBD + CTB group, while the RBD K10 + CTB group demonstrated titers of 1:78, 1:62, and 1:69 at the corresponding time points (Figure 7B). The results demonstrate that the combination of RBD and CTB sustains long-term IgG and IgA responses in serum, with the addition of 10 lysine residues to RBD (RBD K10)-enhancing immunogenicity.

### 3.7. RBD K5 Plus CTB Boosts Immunogenicity via Pulmonary Delivery in Mice

**Figure 8:**
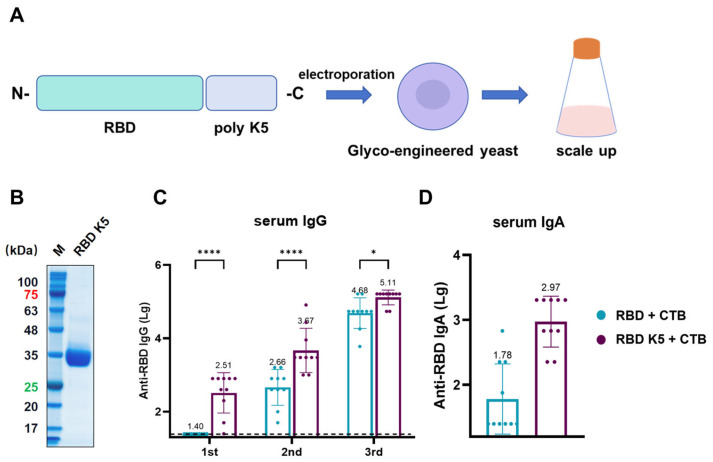
Characterization and immunogenicity of glycoengineered *P. pastoris*-expressed RBD K5 fusion protein. (**A**) Diagrammatic illustration of RBD K5 expression in glycoengineered *P. pastoris*. The RBD K5 fusion construct was incorporated into glycoengineered *P. pastoris* through electroporation, followed by extensive expression on a large scale. (**B**) SDS-PAGE analysis of purified RBD K5 protein. These numbers indicate the molecular weights of proteins, with red indicating a protein molecular weight of 75 kDa and green indicating a molecular weight of 25 kDa. (**C**) Serum IgG levels against RBD at different time points. (**D**) Serum IgA levels against RBD after the third immunization. * *p* < 0.05; **** *p* < 0.0001.

The above findings suggest that incorporating poly-lysine and administering it via pulmonary delivery in mice enhances antigen immunogenicity. To examine the effect of varying the number of lysine residues on immunogenicity, we designed an antigen in which RBD was modified with five lysine residues and assessed its immunological efficacy (Figure 8A). Similar to RBD K10, the constructed plasmid was electroporated into glycoengineered *P. pastoris*. After selecting positive clones and scaling up cultivation, the target protein was purified. SDS-PAGE analysis of the purified protein revealed a target band at approximately 35 kDa (Figure 8B). All mouse groups were immunized via pulmonary delivery on days 0, 14, and 28, with blood samples collected prior to each immunization. For serum IgG (Figure 8C), the RBD K5 + CTB group exhibited a significantly higher antibody titer (1:324) compared to the RBD + CTB group (1:25, *p* < 0.0001) following the first immunization. Following the second immunization, the trend persisted, with the RBD K5 + CTB group exhibiting a significantly elevated antibody titer (4.68 × 10^3^) in comparison to the RBD + CTB group (4.57 × 10^2^, *p* < 0.0001). Post-third immunization, although the disparity in antibody titers between the two groups decreased, the RBD K5 + CTB group (1.29 × 10^5^) continued to demonstrate a significantly higher titer than the RBD + CTB group (4.79 × 10^4^, *p* < 0.05). Furthermore, the serum RBD-specific IgA antibody titer in the RBD K5 + CTB group reached 1:933 (Figure 8D), which was substantially higher than that observed in the RBD + CTB group (1:60, *p* < 0.0001). These findings indicate that the incorporation of five lysine residues into RBD, in conjunction with CTB, induces a more robust immune response.

## 4. Discussion

The incorporation of poly-lysine into the RBD, in conjunction with CTB as a mucosal adjuvant, has been demonstrated to elicit robust mucosal and humoral immune responses in murine models. This approach effectively mitigates the desorption issues associated with RBD and poly-lysine, while maintaining a straightforward preparation process. Another study has employed albumin as a delivery vehicle for RBD antigens [25]; however, albumin is a highly prevalent endogenous protein in the human body, to which the immune system typically exhibits tolerance.

By appending ten lysine residues to the C-terminus of the RBD, the isoelectric point (pI) is increased from 8.73 to 9.47, as calculated using the ExPASy Compute pI/Mw tool. The presence of phospholipid molecules and proteins on the cell membrane surface imparts a negative charge to the membrane. Under physiological conditions, augmenting the number of positively charged residues in the RBD may enhance its interaction with the cell membrane, thereby potentially improving the efficiency of antigen presentation. Previous research has demonstrated that a net positive charge can enhance the binding and internalization of peptides at the cell membrane surface [26]. Positively charged antigens facilitate electrostatic interactions with negatively charged cell surfaces, promoting uptake and immune activation. Modifications in charge may similarly strengthen interactions with cellular components, potentially enhancing vaccine efficacy [27].

Following mucosal immunization in mice, our observations indicated that the mere addition of K10 to the RBD did not significantly enhance its immunogenicity, as depicted in Figure 3B. This lack of enhancement may be attributed to the relatively small molecular weight of the RBD, even with the inclusion of K10, which could result in rapid in vivo clearance or inefficient uptake and processing by APCs. Such limitations may restrict its ability to induce a robust humoral and mucosal immune response. In contrast, the incorporation of the CTB as a mucosal adjuvant significantly augmented the vaccine’s immunogenicity. Notably, the RBD with K10 elicited a more potent immune response. CTB is well-established as an effective mucosal adjuvant, known for its capacity to enhance antigen delivery and stimulate both mucosal and systemic immune responses. It facilitates antigen uptake by binding to GM1 ganglioside receptors on epithelial cells and APCs, thereby promoting antigen presentation and the activation of immune cells such as DCs and B cells [28]. We hypothesis that the enhanced immunogenicity of RBD-K10/K5 is due to improved mucosal uptake and/or APC activation, which indicates that positively charged antigens facilitate electrostatic interactions with negatively charged cell surfaces, thereby promoting uptake and immune activation [27,29]. There may be a synergistic interaction between K10 and CTB, K10/K5’s positive charge enhancing CTB’s mucosal adhesion or influencing APC signaling.

The BALF IgA responses (Figure 5B) were notably weak compared to IgG. The detection of IgA in BALF is often contingent upon the sensitivity of the assay, given that IgA concentrations in mucosal fluids are generally lower and exhibit greater variability compared to IgG [30]. Furthermore, IgA undergoes dilution during BALF collection and possesses a shorter half-life, rendering it less stable at mucosal surfaces due to proteolytic degradation or mucus clearance. In contrast, IgG, which may transude from the serum, demonstrates greater stability [31].

To further examine the influence of lysine residue quantity on immunogenicity, our observations indicate that the addition of five lysine residues (K5) to the RBD produces effects analogous to those observed with the addition of ten lysine residues (K10), as depicted in Figure 8. The incorporation of K5 increases the isoelectric point (pI) of the RBD to 9.20, which, although lower than the pI of 9.47 achieved with K10, still represents a significant shift from the unmodified RBD’s pI of 8.73. This suggests that even a moderate increase in positive charge may be adequate to enhance interactions with the negatively charged cell membrane under physiological conditions. Our findings suggest that incorporating 5–10 lysine residues on the RBD antigen enhances the immune response.

The antigens were synthesized using a yeast expression system optimized through glycoengineering, which enables high-throughput production. The design simplicity is achieved by incorporating lysine residues exclusively on the RBD, ensuring a precise and well-defined antigen composition. This methodology not only streamlines the manufacturing process but also enhances production scalability. The RBD-K10/K5 antigen, produced in glycoengineered yeast cells, demonstrates exceptional formulation stability and scalability, thereby supporting its potential for clinical development. To facilitate translational advancement, we intend to optimize the antigen’s formulation by assessing lyophilization processes and excipient compatibility to ensure long-term stability across various storage conditions. Dose optimization studies will aim to identify the minimal effective doses in preclinical models, balancing immunogenicity and safety, while also exploring alternative delivery methods, such as nasal sprays, to enhance mucosal immune responses. Furthermore, we plan to conduct toxicology studies in larger animal models to evaluate safety profiles and to scale up production in compliance with Good Manufacturing Practice (GMP) standards to satisfy clinical trial requirements.

However, this study has several limitations. Primarily, the current method of mouse immunization involves pulmonary delivery; future research will investigate the administration of the mucosal vaccine via aerosol inhalation in mice. Secondly, the monitoring of vaccine efficacy has been limited to a period of six months following the third immunization. Consequently, it is imperative to extend the surveillance period to comprehensively evaluate the vaccine’s effectiveness. Furthermore, the vaccine’s efficacy has been validated exclusively in BALB/c mice, necessitating further validation in additional animal models, such as hamsters and rhesus macaques. Finally, we will conduct an in-depth investigation into the mechanisms underlying this vaccine design through a series of in vitro experiments, including studies focused on antigen uptake. These considerations highlight the critical need for thorough evaluation and validation prior to clinical translation, thereby ensuring the robustness and applicability of the mucosal vaccine approach.

## 5. Conclusions

In this study, we enhanced the isoelectric point of the target protein by incorporating 10 or 5 lysine residues into the RBD. Upon pulmonary delivery in mice, in conjunction with CTB, this mucosal vaccine induced strong humoral and mucosal immune responses, demonstrated neutralizing antibody activity, and suggested a degree of durability in the immune responses observed.

## Data Availability

The raw data supporting the conclusions of this article will be made available by the authors, without undue reservation.
